# Retracted: Neuropilin-1^high^CD4^+^CD25^+^ Regulatory T Cells Exhibit Primary Negative Immunoregulation in Sepsis

**DOI:** 10.1155/2020/9195623

**Published:** 2020-09-18

**Authors:** Mediators of Inflammation

*Mediators of Inflammation* has retracted the article titled “Neuropilin-1^high^CD4^+^CD25^+^ Regulatory T Cells Exhibit Primary Negative Immunoregulation in Sepsis” [1] due to figure duplication between articles by the same authors and within the article.

Figure duplication concerns were raised to our attention and then noted on PubPeer [2]. A reassessment of the article concluded that a number of panels in Figure 1(e-f) and Figure 2(c) of [1] were duplicated with panels in Figure 2 of [3]. Duplications with another article were also identified [4], where the first, second, and fourth panels of Figure 4(c) are duplicated by the seventh, eighth and ninth panels of Figure 1(e) in [1].

Duplications were also identified within the article [1]:

- Panel 4 in Figure 1(e) is the same as panel 10 in Figure 2(c).
- Panel 1 in Figure 1(f) is the same as panel 5 in Figure 1(f).

The authors did not provide a satisfactory response and the article is therefore being retracted with the agreement of the Chief Editor. The authors do not agree to the retraction.
